# An LC-MS/MS method for the determination of W18 in urine samples

**DOI:** 10.1192/j.eurpsy.2024.464

**Published:** 2024-08-27

**Authors:** R. Quraishi, A. Kumar

**Affiliations:** National Drug Dependence Treatment Centre, All India Institute of Medical Sciences, Delhi, India

## Abstract

**Introduction:**

Synthetic drugs pose one of the most significant drug problems worldwide. In this category, W18 emerges as a potent drug of abuse chemically related to fentanyl. W18 has an analgesic potency 10,000 times greater than morphine. Recent in-vitro studies reported no activity of W18 towards opioid receptors. However, its presence in seized drug samples indicates its use as a precursor in fentanyl synthesis. This emphasizes the need to develop methods for its detection in developing countries dealing with emerging new drugs.

**Objectives:**

To develop an analytical method for the determination of W18 in urine samples.

**Methods:**

Standards with W18 concentrations ranging from 5-500 ng/ml were prepared in negative urine along with deuterated internal standard. The samples were diluted with methanol, centrifuged and the supernatant was subjected to Liquid chromatography-tandem mass spectrometry (LC-MS-MS) with time of flight (QTOF) analysis. For chromatographic separation, a C18 column with 50 degrees oven temperature was used. The mobile phase consists of formic acid, water, and acetonitrile. The TOF MS was operated in positive ion mode and multiple reaction monitoring was used for quantification.

**Results:**

The retention time of W18 was obtained at 9.57 minutes. The parent ion with molecular weight 422.1 along with precursor ions Q1-273, Q2-111.0, Q3-150.0 g/mol were measured. The area of the standards ranges from 1 to 9.0 log 5 with R square of 0.99. The limit of detection (LOD) and quantitation were 5 and 20 ng/ml respectively. The recovery of W18 was estimated to be 96% from the from spiked urine standards.

**Conclusions:**

The developed method is able to detect W18 presence in urine samples. This method has the potential to be used in clinical and research studies.

**Disclosure of Interest:**

None Declared

